# Human Retrovirus Genomic RNA Packaging

**DOI:** 10.3390/v14051094

**Published:** 2022-05-19

**Authors:** Heather M. Hanson, Nora A. Willkomm, Huixin Yang, Louis M. Mansky

**Affiliations:** 1 Molecular, Cellular, Developmental Biology, and Genetics Graduate Program, University of Minnesota—Twin Cities, Minneapolis, MN 55455, USA; hans4784@umn.edu; 2 Institute for Molecular Virology, University of Minnesota—Twin Cities, Minneapolis, MN 55455, USA; willk035@umn.edu (N.A.W.); yang5928@umn.edu (H.Y.); 3 DDS-PhD Dual Degree Program, University of Minnesota—Twin Cities, Minneapolis, MN 55455, USA; 4 Comparative Molecular Biosciences Graduate Program, University of Minnesota—Twin Cities, St. Paul, MN 55455, USA; 5 Masonic Cancer Center, University of Minnesota—Twin Cities, Minneapolis, MN 55455, USA; 6 Division of Basic Sciences, School of Dentistry, University of Minnesota—Twin Cities, Minneapolis, MN 55455, USA

**Keywords:** human retrovirus, RNA encapsidation, nuclear export, RNA dimerization, RNA translocation, lentivirus, deltaretrovirus

## Abstract

Two non-covalently linked copies of the retrovirus genome are specifically recruited to the site of virus particle assembly and packaged into released particles. Retroviral RNA packaging requires RNA export of the unspliced genomic RNA from the nucleus, translocation of the genome to virus assembly sites, and specific interaction with Gag, the main viral structural protein. While some aspects of the RNA packaging process are understood, many others remain poorly understood. In this review, we provide an update on recent advancements in understanding the mechanism of RNA packaging for retroviruses that cause disease in humans, i.e., HIV-1, HIV-2, and HTLV-1, as well as advances in the understanding of the details of genomic RNA nuclear export, genome translocation to virus assembly sites, and genomic RNA dimerization.

## 1. Introduction

Human retroviruses have infected millions of individuals worldwide. Human immunodeficiency virus type 1 (HIV-1, a lentivirus), the most prevalent human retroviral infection, and human immunodeficiency virus type 2 (HIV-2) cause AIDS, which can be treated with life-long antiretroviral therapy. Human T-cell leukemia virus type 1 (HTLV-1, a deltaretrovirus) infects an estimated 15 to 20 million individuals, and HTLV-1 infection can cause adult T-cell leukemia/lymphoma (ATLL) and HTLV-1 associated myelopathy/tropical spastic paraparesis (HAM/TSP). There are no successful antiviral treatments for HTLV-1 infection. Understanding the life cycle of human retroviruses on a molecular level has led to the successful development of the aforementioned antiretrovirals. A further understanding of retroviral replication can expand the development of treatments to combat human retroviral infections, and aid in the development of next-generation antiretroviral therapies.

Retroviruses encode for a positive sense, single-stranded RNA genome. The genome is relatively large, ranging in size from 9 kB to 10.2 kB for HIV-1, HIV-2, and HTLV-1. All human retroviral genomes contain a set of conserved genes, *gag*, *pol*, and *env*. Gag is the main structural protein of retroviruses, Pol is a polyprotein that includes the retroviral enzymes (i.e., protease, reverse transcriptase, integrase), and Env is the attachment protein. Human retroviruses encode a variety of accessory proteins that serve various functions. HIV-1 and HIV-2 encode Tat and Rev while HTLV-1 encodes Tax and Rex to control gene expression. Other accessory genes include those that counteract host restriction factors such as *vif* of both HIV-1 and HIV-2, *vpu* of HIV-1 and *vpx* of HIV-2. HIV-1 and HIV-2 also encode for the accessory genes *nef* and *vpr*. HTLV-1 expresses many accessory genes of various functions through the pX region by alternative splicing. HTLV-1 *hbz* gene encodes for HBZ which plays a major role in HTLV-1 pathogenesis.

Human retroviral genomes also contain noncoding sequences that serve various roles in retrovirus replication. Human retroviral genomes contain long terminal repeats at the 5′ and 3′ ends that contribute to genome replication and integration into the host genome. The 5′ untranslated region of retroviral genomes contains a region of high secondary structure and contains specific structures that mediate dimerization and genome packaging. Human retroviruses encode a secondary structural element that mediates export of retroviral unspliced genomic RNA from the nucleus, i.e., the Rev responsive element (RRE) (for HIV-1 and HIV-2) and the Rex responsive element (RxRE) (for HTLV-1).

The retroviral genome is converted to a double-stranded viral DNA. Recent evidence suggests that capsid core uncoating and reverse transcription primarily occur in the nucleus [1,2]. The cDNA is integrated into the host genome and host cell machinery transcribes the provirus to make new full-length genomic RNA (gRNA) copies and differently spliced transcripts. In order to assemble new viral particles containing the retroviral gRNA, these viral RNA transcripts must be exported from the nucleus. This requires a specialized mechanism to export the unspliced gRNA. The exported gRNA is then translocated to virus assembly sites at the plasma membrane. During virus particle assembly, two copies of noncovalently linked gRNA are packaged. The process of gRNA packaging remains incompletely understood for human retroviruses. Here, we review three key processes required for infectious particle assembly containing gRNA—(a) gRNA nuclear export, (b) gRNA translocation to particle assembly sites, and (c) gRNA packaging/encapsidation.

## 2. Genomic RNA (gRNA) Nuclear Export

In order to be packaged at the plasma membrane into virus particles, the unspliced retroviral gRNA must be exported from the nucleus despite not being recognized by standard RNA nuclear export machinery. Two cellular pathways have been identified to be hijacked by retroviruses to export unspliced and partially spliced viral RNAs from the nucleus. Some retroviruses utilize the TAP/NXF1 pathway, while other retroviruses have been found to use the CRM1/XPO1 nuclear export receptor. For human retroviruses, HIV-1, HIV-2 and HTLV-1, gRNA nuclear export utilizes the CRM1 pathway [3,4,5,6,7,8,9,10,11,12]. These complex human retroviruses encode accessory proteins that interface with the CRM1 export machinery. HIV encodes the accessory protein Rev, which mediates post-transcriptional gene expression [13,14,15]. HTLV-1 encodes for a homologous protein, Rex, that is required for post-transcriptional viral gene expression [16]. Both Rev and Rex permit retroviral gene expression by directly mediating the export of unspliced or partially spliced viral RNAs [15,17,18,19,20], therefore indirectly contributing to gRNA packaging.

### 2.1. Retroviral Rev and Rex Accessory Proteins Shuttle between the Nucleus and Cytoplasm

**Figure 1:**
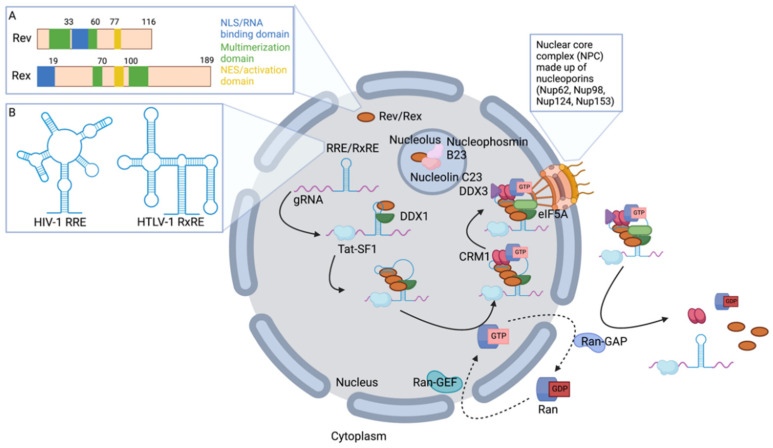
Nuclear export of human retroviral gRNA. (**A**)**.** The mapped domains in the HIV-1 Rev and HTLV-1 Rex proteins. Nuclear localization signal (NLS) Nuclear export signal (NES) (**B**). The secondary structure of the HIV-1 RRE and HTLV-1 RxRE. Rev responsive element (RRE) Rex responsive element (RxRE) See text for further details. Note that the RRE and RxRE structures are not drawn to scale. DEAD box helicase 1 (DDX1), DEAD box helicase 3 (DDX3), HIV Tat-specific factor 1 (Tat-SF1), Chromosomal Maintenance 1, also known as Exportin 1 (CRM1), Ras-related Nuclear protein (Ran), Ran Guanine nucleotide exchange factor (Ran-GEF), Ran GTPase activating protein (Ran-GAP), Eukaryotic translation initiation factor 5A (eIF5A). Created with BioRender.com software (accessed on 4 May 2022).

Rev and Rex are nucleocytoplasmic shuttle proteins. The shuttle function of the Rev and Rex proteins is mediated by a nuclear localization signal (NLS) and a nuclear export signal (NES) that is encoded within each protein; the NES is necessary for retroviral gRNA nuclear export [21,22,23]. The nuclear import of Rev and Rex allows for the interaction of these proteins with their respective unspliced gRNA in the nucleus. Rev nuclear import alone is not sufficient for HIV gRNA nuclear export, and retroviral gRNA nuclear export also depends on the nucleolar localization of the Rev [24,25] and Rex proteins [26] (Figure 1). Basic residues in the Rev and Rex RNA-binding domains mediate nucleolar localization [23,27,28]. While the significance of Rev and Rex nucleolar localization in facilitating gRNA nuclear export remains poorly understood, it is thought that nucleolar localization allows Rev/Rex interaction with host cell proteins involved in nuclear export of unspliced retroviral RNAs. For example, a recent study employing immunoprecipitation and mass spectrometry identified several nucleolar factors that interact with Rev only when Rev nucleolar localization sequences remain intact [29] (Figure 1). This observation suggests that proteins including nucleophosmin B23, nucleolin C23, and various cellular splicing factors interact with HIV-1 Rev in nucleoli and may impact gRNA nuclear export.

NES-mediated nuclear export of Rev (HIV-1, HIV-2) and Rex (HTLV-1) drives the export of retroviral RNAs. The NES is located within the activation domain of Rev and Rex proteins and contains numerous leucine residues associated with the nuclear export function [21,23,27,30,31]. Several studies have demonstrated that swapping homologous domains between Rev and Rex can result in the export of HIV or HTLV gRNAs [32,33,34,35,36]. These functional chimeras suggest a partially conserved mechanism of gRNA export. The observation of nonfunctional swaps between Rev and Rex nucleolar-targeting sequences [36] underscores potential differences in their mechanism of gRNA nuclear export. Investigation of these differences will help delineate the mechanism(s) of unspliced gRNA nuclear export.

### 2.2. Rev and Rex Interact Directly with gRNA

Retroviral accessory protein-mediated export of unspliced gRNAs occurs through direct RNA-protein interactions that form the export complex, and arise cotranscriptionally, before spliceosome activation [37]. The timing of complex formation allows for export of unspliced retroviral gRNA before it is processed. In the case of HIV-1, Rev specifically interacts with the RRE in the HIV-1 gRNA [38,39,40]. The HIV-1 RRE is a 530 base-pair sequence located in the *env* region of the genome and is retained in unspliced and partially spliced viral RNA species. The RRE has a complex RNA secondary structure comprised of a central stem and 5 stem-loop structures [17,41,42] (Figure 1). Several reports have indicated the requirement of the RRE secondary structure for Rev binding and have shown that the initial Rev-RRE interaction occurs at a high affinity binding site, i.e., stem-loop IIB [43,44,45,46]. Rev binds the RRE through an arginine-rich RNA binding domain contained within an alpha-helical structure [24,47,48,49,50]. This interaction between Rev and RRE is the foundation of the nuclear export complex.

Interactions between viral accessory proteins mediating nuclear export and the corresponding RNA sequence element are similar among the human retroviruses. The HIV-2 genome also contains a RRE (RRE-2) located in the *env* region of the genome that has complex secondary structure [42,51,52,53]. Like Rev, Rex also interacts with a specific RNA sequence to permit nuclear export, i.e., the RxRE [54,55,56,57]. Like the RRE, the RxRE has complex secondary structure, specifically it is made up of a long stem and four stem-loop structures [55,58,59] (Figure 1). The role of the stem-loop structures of the RxRE has been investigated in regards to their importance in RNA export efficiency [4,60]. For Rex, residues similar to those involved in Rev-RRE binding are required for RxRE binding. The RNA-binding domain of Rex is rich in basic residues, and specific residues important for binding have been mapped [32,57,61]. The interaction of HIV-2 Rev and HTLV-1 Rex with RRE-2 or RxRE containing RNAs, respectively, have not been studied as extensively as the interaction of HIV-1 Rev and RRE-1.

These similarities between Rev and Rex, and their corresponding target sequences, imply that they mediate nuclear export through a conserved mechanism. Rex has been shown to export RNAs containing RRE-1 and RRE-2 from the nucleus, but Rev cannot function in the export of RxRE-containing RNAs [62,63,64,65]. Some conservation of function has also been observed between HIV Rev and RRE sequences. For example, HIV-1 Rev can export unspliced viral RNAs containing the RRE-2, but HIV-2 Rev cannot mediate export of RRE-1 containing RNAs [52,64,65,66,67]. Further comparative analysis between human retroviruses that explore the incomplete conservation of Rev and Rex function is likely to reveal further details on the mechanism(s) of retroviral gRNA nuclear export.

The Rev-RRE interaction induces structural changes of RRE-containing RNAs that promote nuclear export. Specifically, initial Rev binding results in changes in the stem-loop structures of RRE-containing RNAs that allows for further Rev binding [68]. Sequential, cooperative binding of Rev to adjacent low affinity binding sites along the RRE results in Rev oligomerization [47,69,70,71,72,73] (Figure 1). Dissociation constants have been reported for Rev-RRE binding as 0.26–33 nM for Rev monomers and 0.77–92 nM for Rev dimers [71,72]. Up to 6–8 Rev molecules have been reported to bind the RRE in vitro [73]. Several studies have reported mutant Rev proteins that prevent oligomerization and do not bind RRE-containing RNAs, which impedes gRNA nuclear export [47,69,74,75,76]. This demonstrates the importance of Rev oligomerization in retroviral gRNA nuclear export and indicates the requirement of both protein-protein and protein-RNA interactions in this process. While Rev oligomerization is mediated by cooperative binding to RNA, residues of the arginine-rich RNA binding domain are not implicated in Rev oligomerization. In contrast, residues proximal to this region that comprise a hydrophobic patch on the surface of Rev are necessary for oligomerization [5,69,75,77]. Rev oligomerization is structurally linked to the specificity of the Rev-RRE interaction. This conclusion is supported by the solved structure of a Rev dimer that can bind the RRE, and by the extent to which HIV-1 and HIV-2 Rev can function with the opposite RRE—i.e., HIV-1 Rev can export HIV-2 gRNA, but HIV-2 Rev cannot export HIV-1 gRNA [66,78,79]. Rex oligomerization is required for export of HTLV-1 gRNA [80]. However, the HTLV-1 gRNA export mechanism is not as completely characterized as Rev oligomerization. Rev and Rex oligomerization are required for interaction with the CRM1 nuclear export pathway.

### 2.3. Host Proteins Involved in gRNA Nuclear Export

Cellular proteins have been shown to be involved in Rev binding to the RRE. CRM1 is a major nuclear export receptor that interfaces with the Rev-RRE complex and permits incompletely spliced RNA export from the nucleus. CRM1 interacts directly with Rev as a dimer [81] (Figure 1 and Table 1), and Rev serves as an adaptor to the cellular pathways that are hijacked by retroviruses to export gRNA. CRM1-mediated export of gRNA requires RanGTP interaction with the Rev-CRM1 complex [7,82]. Ran proteins are small G proteins that mediate translocation of cellular proteins and RNA through the nuclear pore complex (NPC). Ran proteins bind GTP in the nucleus and GDP in the cytoplasm, due to Ran GAPs that are primarily localized to the cytoplasm and Ran GEFs that are localized in the nucleus. RanGTP-bound CRM1 directs the Rev-RRE ribonucleoprotein complex out of the nucleus through direct interaction with the mRNA translocation machinery located at the nuclear envelope [3] (Figure 1 and Table 1). The GTP hydrolysis drives export of the viral gRNA utilizing the Ran cycle. The RanGTP hydrolysis in the cytoplasm dissociates the export complex.

DEAD box helicase (DDX) proteins interact with RRE-Rev-CRM1 complex to promote nuclear export (Figure 1 and Table 1). The DDX1 protein acts as a clamp on the RNA to nucleate oligomerization by promoting binding of the first Rev monomer to the RRE [83,84]. A recent study has further clarified the role of DDX1 in nucleating Rev oligomerization by showing that despite tighter binding to Rev, DDX1 nucleation of oligomerization is mediated through interaction with the RRE [84]. In particular, this study showed that a DDX1 mutant that cannot bind Rev can still nucleate oligomerization, and that DDX1 mutations that reduced the ability to bind RNA disrupted the ability of DDX1 to promote the first Rev monomer binding to gRNA [84]. The interaction of DDX1 with RRE RNA results in a structural change of the RRE RNA at stem IIB that enhances Rev binding and oligomerization [85]. DDX1 is hypothesized to either promote this structural change and/or stabilize it. DDX3 has been hypothesized to restructure retroviral gRNA in order to make it amenable to translocation through the nuclear pore by interaction with CRM1 [86,87] (Figure 1 and Table 1). The contribution of DDX1 and DDX3 is complicated by the conservation of other DEAD-box helicases which could permit interaction with the RRE. It is plausible that this effect could be the result of several DEAD-box helicases. 

Additional cellular proteins interact with the Rev-CRM1 complex and have been shown to contribute to export of retroviral RNAs. Phosphofurin acid cluster sorting protein 1 (PACS1), which localizes furin to the trans-Golgi network, can also shuttle between the nucleus and cytoplasm, can associate with Rev and CRM1, and can contribute to nuclear export of viral transcripts (Table 1) [88]. Moreover, PACS1-mediated export of viral transcripts was discovered to be due to interaction with the Rev-CRM1 pathway [88]. Other proteins, such as acidic leucine-rich nuclear phosphoprotein 32 family member A and B (i.e., ANP32A, ANP32B), mediate the export of unspliced or partially-spliced viral mRNA via interactions with Rev and CRM1 (Table 1) [89]. In particular, a double knockout of ANP32A and ANP32B led to a significant decrease in Gag expression and a dramatic accumulation of unspliced viral mRNAs in the nucleus, while reconstitution of either ANP32A or ANP32B restored virus replication [89]. 

The Rev-RRE-CRM1 nuclear export complex interacts with the translocation machinery which is comprised of various cellular proteins. Some aspects of the NPC implicated in HIV-1 gRNA export have been characterized. Nup98, a nucleoporin, has been shown to interact with Rev, which implies it is likely part of the NPC responsible for Rev-mediated RNA export (Table 1) [90]. Other nucleoporins implicated in retroviral gRNA nuclear export include Nup124, Nup153, Nup98, and Nup62 (Table 1) [91]. Interaction of the Rev-RRE-CRM1 complex with the NPC is mediated by eIF5A [8,91,92] (Figure 1 and Table 1). It is known that eIF5A is essential for directing the retroviral export complex to the translocation machinery. This observation was made in cell-free studies, and it is possible that several DDX proteins may be contributing to restructuring of the retroviral gRNA that promotes nuclear export in cells. Part of the translocation machinery involved in export of HIV and HTLV gRNAs include nucleoporin-like protein Rab/hRIP, which interacts with CRM1 (Table 1) [31,93,94,95]. The specific function of Rab/hRIP in the nuclear export machinery of retroviral gRNA is not fully understood. Collectively, the interactions between cellular proteins and the export complex result in the transport of Rev and Rex with RRE- or RxRE-containing RNA out of the nucleus as a stable complex with CRM1. Studies with HIV-1 have provided much of the current understanding of the role of CRM1 and other associated cellular proteins in viral RNA transport. The previously described similarities in the viral components of nuclear export suggest potential overlap in the mechanisms of nuclear export among the human retroviruses. Comparable studies with HIV-2 and HTLV-1 regarding the involvement of other components of the translocation machinery are lacking.

Other cellular proteins that have been shown to be involved in the export of HIV-1 gRNA include UPF1, MOV10, Staufen2 and Tat-SF1. Host upframeshift protein 1 (UPF1) regulation of viral RNA nuclear export relies on the nucleocytoplasmic shuttling of UPF1 [96]. UPF1 exists in two essential viral ribonucleoprotein (RNP) complexes during the late phase of HIV-1 replication. The first type of RNP is nuclear export RNPs that contain Rev, CRM1, DDX3, and the nucleoporin p62; the second type of RNA is cytoplasmic RNPs that exclude these nuclear export markers but contain Gag (Table 1) [96]. UPF2 was found to be excluded from the UPF1-Rev-CRM1-DDX3 complex and was identified as a negative regulator of viral RNA nuclear export [96]. The RNA helicase MOV10, a member of the UPF1-like superfamily, was found to facilitate Rev/RRE-dependent nuclear export of viral RNAs, as a co-factor of HIV-1 Rev (Table 1) [97]. MOV10 interacts with Rev in an RNA-independent manner as determined by co-immunoprecipitation analysis [97]. The DEAG (Asp-Glu-Ala-Gly) box of MOV10 is required to enhance Rev/RRE-dependent nuclear export [97]. Staufen2 positively regulates Rev nuclear export, which is predicted to contribute to gRNA nuclear export [98]. Lastly, human Tat-specific factor 1 (Tat-SF1) was found to bind the HIV-1 genome at the trans-acting response (TAR) sequence and selectively transport HIV-1 RNAs by facilitating the nuclear export of unspliced gRNAs, while retaining singly spliced viral RNAs in the nucleus (Table 1) [99]. Cell-free studies have revealed some details of the Tat-SF1 interaction with the HIV-1 genome [99]. In particular, Tat-SF1 forms a complex with the TAR RNA independent of Tat. Tat-SF1 interacts with at least one additional location in the 5′ end of the HIV-1 gRNA, and this interaction is enhanced by Tat [99]. Taken together, these findings suggest that nuclear export of retroviral gRNA is a highly regulated process requiring several other cellular proteins. Future studies into how these cellular proteins interface to coordinate retroviral gRNA nuclear export will provide a more complete understanding of the mechanism(s) of RNA export. Such studies have yet to be fully extended to other human retroviruses.

A role for Gag in retroviral gRNA nuclear export has been suggested. For example, several retroviral Gag proteins, including those of Rous sarcoma virus (RSV) and HIV-1, have been observed in the nucleus, though the nuclear roles of Gag are not fully characterized. The RSV Gag protein interacts with unspliced viral RNA in the nuclei of infected cells to form viral RNPs [100]. RSV viral RNPs have been implicated in genomic RNA packaging [100]. HIV-1 Gag was also discovered to form viral RNP complexes with unspliced viral RNA at transcription sites [101]. The interaction of nuclear HIV-1 Gag with unspliced gRNA is specific and has been visualized in discrete foci in cell nuclei. Three-dimensional imaging analysis has revealed that HIV-1 Gag was localized to the perichromatin space and associated with both unspliced gRNA and Rev in a tripartite RNP complex. This observation supports a model where Gag interacts with newly transcribed gRNA to form nuclear RNP complexes which may aid in export and/or packaging.

## 3. Translocation of gRNA to the Plasma Membrane

After the gRNA is exported out of the nucleus, the gRNA translocates through the cytoplasm to sites of virus particle assembly at the plasma membrane. One study reported tracking many individual HIV-1 gRNA molecules in the cytoplasm, and found most gRNAs moved in a manner that was ‘nondirectional’ and ‘random walk-like’, indicating that the HIV-1 gRNA moves by diffusion throughout the cytoplasm [102]. This type of gRNA movement is observed whether HIV-1 Gag is present or not [102]. Diffusive movement implies that Gag does not guide the movement of HIV-1 gRNA in a directed manner nor do cellular proteins. Furthermore, the observation of single HIV-1 gRNA molecules over time has revealed that at different time points, a given HIV-1 gRNA molecule could move at various speeds [102]. This change in gRNA movement implies that the local gRNA environment dictates gRNA behavior. These data, taken together, suggest that retroviral gRNA is not directly transported to virus assembly sites by a single mechanism and that several host cell components locally contribute to gRNA translocation to virus assembly sites. While the studies described in this review suggest various ways in which viral or host cell components may contribute to gRNA translocation to virus assembly sites, a cohesive model for retroviral gRNA translocation to assembly sites is lacking. The importance of all these factors in the context of retroviral infection events in infected individuals is unclear.

### 3.1. Influence of Nuclear Export Pathway on gRNA Translocation to the Plasma Membrane

Components of the nuclear export machinery have been implicated to influence gRNA translocation to virus particle assembly sites. For instance, changing the RNA export pathway from RRE-dependent trafficking via CRM1 to constitutive transport element (CTE)-dependent export via NXF1 was necessary for HIV-1 replication in murine cells [103]. The requirement of a specific nuclear export pathway in murine cells would imply a link between that pathway and gRNA translocation to virus assembly sites. In a system with HIV-1 gRNAs exported by different nuclear export pathways (i.e., CRM1 or NXF1), the gRNAs exported by different pathways were not copackaged [104]. Comparing retroviral gRNA exported by different pathways can provide further insights into potential linkage(s) of these processes. For example, HIV-1 gRNAs exported by the CRM1 or NXF1 pathway were found to have diffusive movement in the cytoplasm, while CTE-containing RNAs diffuse more slowly than RRE-containing RNAs [105]. While the biology causing observed differences in diffusion rates is unknown, one possible explanation is that RNAs exported by different pathways are associated with different proteins that alter diffusion rates to varying extents. In contrast, other studies using an HIV-1 RRE-containing RNA observed export to the cytoplasm that occurred in short bursts, while a CTE-containing Mason-Pfizer monkey virus (MPMV) gRNA, viral RNA export was observed to be continuous [106]. The subcellular localization of viral RNAs also differs depending upon the mode of RNA export, with HIV-1 RRE RNAs being exported throughout the cytoplasm with no specific localization, while MPMV gRNAs are specifically exported to the microtubule organizing center (MTOC) [106]. These observed differences in gRNA localization were reversed when the HIV-1 gRNA export pathway was reversed by replacement of the RRE with the CTE [106]. Taken together, the nuclear export pathway could, to some extent, impact viral gRNA localization. For example, components of either system could serve as a signal for a particular cytoplasmic fate. However, one study reported that changes in HIV-1 gRNA localization due to the nuclear export pathway do not change gRNA localization and that the HIV-1 gRNA was not targeted to centrosomes [105]. The differences in reported observations may involve the use of proviruses to express viral components versus transfected DNAs. To date, the precise role of nuclear export in retroviral gRNA translocation to virus particle assembly sites remains poorly understood for HIV-1, let alone HIV-2 and HTLV-1. 

### 3.2. Influence of Gag on gRNA Translocation to the Plasma Membrane

**Figure 2:**
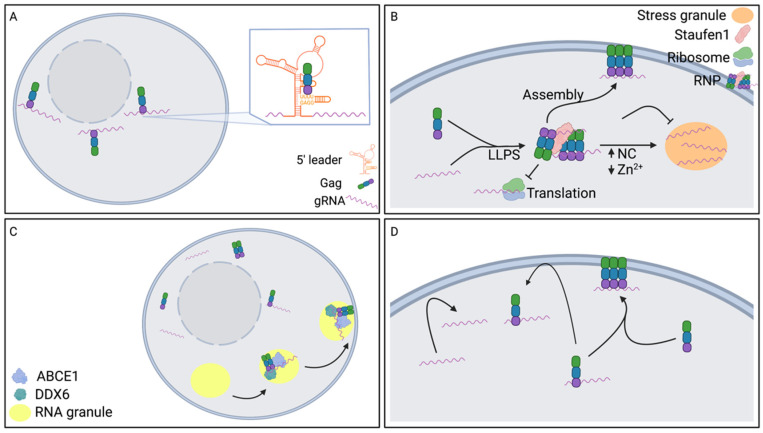
HIV-1 gRNA interaction with Gag and translocation to assembly sites. (**A**). Genome recognition occurs in the perinuclear region of the cell. (**B**). Phase separation of gRNA regulates translocation to virus particle assembly sites. Liquid-liquid phase separation (LLPS), Ribonucleoprotein complex (RNP), Gag nucleocapsid domain (NC). (**C**). Localization of gRNA to assembly intermediates promotes virus particle assembly. DEAD box Helicase 6 (DDX6), ATP Binding Cassette Subfamily E Member 1 (ABCE1) (**D**). Retention of gRNA at the plasma membrane is dependent upon interaction with Gag. Created with BioRender.com (accessed 4 May 2022).

Gag plays a major role in specific gRNA packaging; therefore, it is plausible that Gag may influence gRNA translocation to assembly sites. Genome recognition of gRNA by Gag represents the initial RNA-protein interaction. This process involves HIV-1 Gag specifically interacting with the gRNA in the 5′ region of the genome [107,108]. One study found that HIV-1 Gag and the gRNA colocalized at areas surrounding the nuclear envelope at early time points after the appearance of Gag expression, where this colocalization was shown to be driven by Gag [109,110]. These data, taken together, imply that HIV-1 genome recognition occurs in the perinuclear region of the cell (Figure 2A). However, it is technically challenging to detect single Gag molecules, and therefore the first Gag and gRNA interaction has not been observed in cells, though cell-free studies have detailed HIV-1 genome recognition. For example, a recent study demonstrated that out of more than two dozen binding sites in the 5′ leader sequence of the HIV-1 gRNA, the initial Gag-RNA contact determined by assessing NC binding to a portion of the 5′ leader sequence, occurred at high-affinity binding sites with the UUUU:GGAG motif [111] (Figure 2A). Initial binding of Gag to gRNA is dependent on the structural lability of this motif [111]. Gaining greater insights into the subcellular location of genome recognition will provide new details regarding the role of Gag in gRNA translocation to the plasma membrane. 

Recent studies using transmission electron microscopy (TEM) to study HIV-1 Gag and gRNA localization at high resolution revealed colocalization mainly in the cytoplasm with few instances of colocalization in the nucleus [112]. The site of genome recognition was not defined, and clustering of HIV-1 Gag and gRNA in the cytoplasm was dependent on regions of Gag and gRNA previously reported to be involved in Gag and gRNA interactions [112]. Furthermore, amino acid substitutions at residues shown to reduce gRNA packaging were observed to also reduce Gag and gRNA clustering, implying that Gag and gRNA colocalization in the cytoplasm is important for genome recognition [112]. The relatively high resolution of the TEM approach allowed for measurements of the distance between Gag and gRNA and revealed that the distance between Gag and gRNA was reduced over time. This suggests that Gag and gRNA condense in the cytoplasm [112]. Additional high-resolution studies of Gag and gRNA in the cytoplasm have increased our understanding of translocation of gRNA to the plasma membrane. For example, one study that utilized 3D-super-resolution structured illumination microscopy detected gRNA dimers in the cytoplasm and found that Gag is involved in gRNA dimerization in the cytoplasm [113]. These results help to support the conclusion that Gag is involved in targeting gRNA dimers to the plasma membrane [113]. This study and another reported that Gag interaction with gRNA in the cytoplasm and translocation to the membrane is dependent on the NC domain of Gag and more specifically the zinc-coordinating finger (ZnF) domains [113,114]. Another study supports the model that Gag-gRNA interactions in the cytoplasm promote assembly given that reduction in Gag oligomerization in the cytoplasm occurred when the NC domain was mutated, and Gag-gRNA binding was inhibited [115].

Phase separation of Gag and gRNA in the cytoplasm has recently been reported, where the ZnF domains of HIV-1 Gag NC promote gRNA liquid-liquid phase separation (LLPS) into RNP complexes [116]. These RNPs were theorized to stimulate virus assembly by inhibiting translation and promoting formation of assembly intermediates (Figure 2B). HIV-2 and HTLV-1 Gag may create similar complexes as their respective NC domains also have the ability to phase separate in a Zn^2+^ dependent manner [116]. This study showed that Zn^2+^ concentration can regulate RNP formation through experiments in which high levels of HIV-1 NC resulted in a switch from RNP formation to Zn^2+^ dependent stress granule formation, and in which Zn^2+^ depletion led to gRNA localization to stress granules or retention in the nucleus [116] (Figure 2B). The switch between RNP and stress granule phase separation suggests a potential mechanism that indirectly regulates gRNA trafficking to virus assembly sites controlled by the NC domain of Gag and Zn^2+^. The level of Gag expression and Zn^2+^ containing NC maintains Gag and gRNA RNPs that promote translocation to assembly sites which likely occurs through assembly intermediates (Figure 2B). A key cellular protein, Staufen-1, plays a role in this switch mechanism. Staufen-1 has been shown to associate with HIV-1 gRNA and Gag RNPs as well as promote assembly and gRNA packaging [117,118]. Furthermore, Staufen-1 has been shown to have a role in dissociating HIV-1 stress granules and specifically preventing gRNA localization to stress granules [119] (Figure 2B). HIV-2 gRNA has also been shown to phase separate in RNPs and stress granules. However, the composition of the RNPs and the regulation of phase separation differ from that of HIV-1. While HIV-1 abrogates stress granule formation, HIV-2 infection has been shown to induce stress granule formation. HIV-2 gRNA phase separates into RNPs with a stress granule assembly protein, TIAR, that is found in the cytoplasm or in stress granules [120]. Phase separation of TIAR with HIV-2 gRNA was not dependent on the interaction with Gag, but increased Gag expression promoted stress granule formation [120]. The correlation between a threshold level of Gag expression and gRNA localization to stress granules suggests this may regulate the switch from translation to assembly, and the switch could promote gRNA translocation to assembly sites.

Similar to Gag and gRNA phase separation, Gag and gRNA colocalization in assembly intermediates suggests specific trafficking mechanisms of gRNA prior to localization to assembly sites at the plasma membrane. The HIV-1 gRNA has been reported to colocalize with Gag in complexes larger than 30S, such as Gag assembly intermediates [121]. In particular, HIV-1 gRNA was detected with Gag in an assembly intermediate derived from host RNA granules that contain two cellular proteins that facilitate assembly, ABCE1 and DDX6, an RNA granule protein [121] (Figure 2C). The localization of gRNA to Gag assembly intermediates may suggest a potential gRNA translocation mechanism in which after incorporation to assembly intermediates, the gRNA is translocated to plasma membrane assembly sites by the assembly intermediates.

Behavior of RNA near the plasma membrane can also provide insights into the mechanism of translocation to assembly sites. The HIV-1 gRNA can be localized to the plasma membrane in the absence of Gag and is only transiently near the membrane [107,122] (Figure 2D). The retention of gRNA at the plasma membrane requires Gag and Gag-gRNA interactions via the RNA packaging signal [107,110,122] (Figure 2D). Not all RNAs localized near the plasma membrane are packaged into particles [122]. As Gag expression level increases, the frequency of RNA packaging also increased [122], suggesting that Gag likely plays an important role late in the translocation to a virus assembly site. Gag membrane binding and multimerization are required to retain gRNA at the membrane [107]. It is believed that Gag oligomers can readily recruit gRNA to the plasma membrane for gRNA packaging [107] (Figure 2D). It remains unclear how all the observations described above contribute to gRNA translocation to virus assembly sites in the formation of infectious virus particles.

At the plasma membrane, the gRNA promotes particle assembly. Not only can the HIV-1 gRNA stimulate particle assembly, but the gRNA can stimulate particle production by specific Gag-gRNA interactions [123,124,125] and highlights the importance of specific genome recognition in virus particle production [125]. This further supports studies that have shown that RNA incorporation in virus particles is necessary for virion integrity [126,127,128] and that specific interactions between dimeric HIV-1 gRNA and Gag act to nucleate the process of HIV-1 particle assembly. Retroviral gRNA is particularly important for nucleation at low Gag levels in cells [129], which is thought to be driven by the decrease in activation energy needed for Gag interactions with the gRNA packaging signal that allow for selective packaging. 

### 3.3. Role of Microtubule Organizing Center (MTOC) on gRNA Translocation to the Plasma Membrane

HIV-1 gRNA sequences suggest a role for a cellular protein in translocating gRNA via the MTOC. For example, the HIV-1 gRNA contains two sequences predicted to be compatible for interaction with host protein hnRNP A2, similar to the hnRNP A2 response element (A2RE) found in the human genome [130]. The A2RE-1 site in the HIV-1 gRNA is located in the *gag* gene, while the A2RE-2 site is located in the overlap between the *tat* and *vpr* genes [130]. These sequences are involved in RNA transport in oligodendrocytes [130]. However, the extent of the function of the A2RE-like sequences in the HIV-1 genome is poorly understood. Depletion of hnRNP A2 in HIV-1 expressing cells was found to result in HIV-1 gRNA accumulation at the MTOC [131]. Other studies have found HIV-1 Gag and gRNA colocalization at the MTOC [109]. The accumulation at the MTOC in the absence of hnRNP A2 implies that hnRNP A2 may aid in translocating gRNA to virus assembly sites along microtubules. Alternatively, translocation to the MTOC could provide an opportunity for interaction with other proteins that coordinate trafficking to assembly sites and not via microtubules. Treatment of cells with microtubule depolymerizing drugs was observed to not alter the random-walk like motion of HIV-1 gRNA in the cytoplasm [102], suggesting that the role of hnRNP A2 may be in promoting colocalization with other proteins that permit trafficking to the plasma membrane. No reports of hnRNP A2 in gRNA translocation have been reported for HIV-2 or HTLV-1. The function of gRNA localization to the MTOC remains unclear.

### 3.4. Role of Endosomal Vesicles on gRNA Translocation to the Plasma Membrane 

Endosomal vesicles are another component of the cell that have been reported to influence retroviral gRNA translocation. In a Gag dependent manner, HIV-1 gRNA has been reported to be transported via endosomal vesicles, where the RNA displayed directed movement instead of diffusive cytoplasmic movement for a minority of the gRNA molecules that were tracked [102,132]. It has also been suggested that Gag and gRNA may be transported to the perinuclear region by late endosomes in a dynein dependent manner [133]. This localization and transport via endosomes were not the result of endocytosis of either gRNA or Gag at the plasma membrane [133], suggesting a specific population of gRNA and Gag associates with late endosomes and is packaged into virus particles. 

## 4. Packaging of Human Retroviral gRNA

Retroviral gRNA is specifically packaged as a noncovalently linked dimer. Compared to spliced viral RNAs, HIV-1 gRNA is enriched in virions by a factor of 20 [134]. It has been reported that dimerized gRNA is the RNA unit recognized for gRNA packaging [135,136]. This is supported by studies that show that mutations that promote dimerization between RNAs promote the packaging of those RNAs [135,137]. Inversely, it has been reported that gRNA mutations that prevent dimerization will inhibit gRNA packaging [138,139]. However, one study that made different mutations to prevent dimerization did not find a reduction in packaging, while another study uncoupled gRNA dimerization and packaging and observed robust packaging of gRNA monomers [140,141]. The apparent discrepancies are likely due to the use of different dimerization mutants. For HTLV-1 mutations that disrupted HTLV-1 gRNA dimerization in vitro had no effect on gRNA packaging into released particles [142]. 

Two key interactions are required for specific packaging of dimerized gRNA: 1. A high-affinity protein-RNA interaction between Gag and gRNA and 2. A specific interaction of structural motifs in two gRNA molecules (dimerization).

### 4.1. gRNA Sequences Involved in the Gag-gRNA Interaction

In order to package retroviral gRNA, the Gag protein interacts with a specific *cis*-acting element of the gRNA. This element has been referred to by different names, including the RNA packaging signal (psi) or the RNA encapsidation signal (E). Interaction of Gag with the RNA packaging signal promotes selective packaging of retroviral gRNA over other virus-derived RNAs and cellular RNAs [126,129,143,144]. The RNA packaging signal for retroviruses is comprised of a complex secondary (and tertiary) structure commonly involving several RNA stem-loops. This RNA packaging signal is conserved on a structural level [145,146]. The RNA secondary structure of the stem-loops is important for gRNA packaging [141]. It has been hypothesized that selective packaging by the RNA packaging signal is due to Gag nucleation occurring at a faster rate on these sequences.

**Figure 3:**
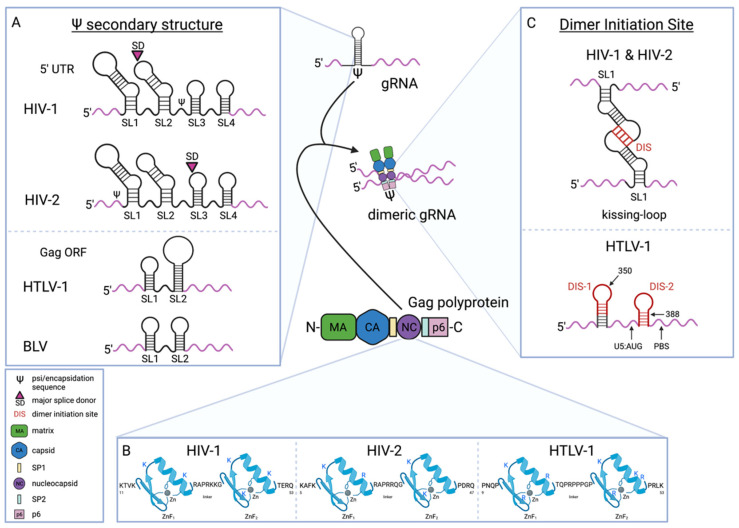
Schematic representation of the human retroviral gRNA packaging process. Gag stabilization of retroviral genome dimers which occurs between two Gag-gRNA duplexes. (**A**) HIV-1 and HIV-2 RNA secondary structure models of the 5-untranslated region (5′ UTR) containing the psi (Ψ) sequence with indication of the major splice donor site (SD) either upstream to the Ψ sequences necessary for packaging, as in HIV-1 or downstream to the Ψ packaging sequences as in the case of HIV-2; HTLV-1 and BLV RNA secondary structure models of the gRNA packaging sequence are located in the Gag open reading frame (ORF). The RNA structural elements are marked as indicated: SL1, stem-loop 1; SL2, stem-loop 2; SL3, stem-loop 3; SL4, stem-loop 4; gRNA, viral genomic RNA. Stem-loops are not drawn to scale. (**B**) Model of the structural motifs in the retroviral Gag polyprotein nucleocapsid (NC) domain mediating the specific Gag-gRNA interaction. Shown are the two zinc-finger (ZnF_1_ and ZnF_2_) motifs in HIV-1, HIV-2, and HTLV-1 NC (amino acid residues 11–53, 5–47, and 9–53 respectively). During HIV-1 gRNA packaging, both ZnFs are necessary as the predominant interacting partners with the gRNA, yet the N-terminal ZnF has a greater impact on gRNA packaging. Highlighted in blue are the specific, positively charged, basic amino acid residues within the two ZnF motifs that have been identified to be involved in binding the gRNA. (**C**) Representative kissing-loop structure induced by NC of HIV-1 and HIV-2 at the gRNA dimer initiation site (DIS), a palindromic sequence located in SL1 of the Ψ sequence secondary structure in the 5′ UTR; representative structure of the two DIS sites identified for HTLV-1 containing palindrome-like sequences located within loop structures in the 5’ leader sequence upstream of the primer binding site (PBS). Created with BioRender.com software (accessed 4 May 2022).

Retroviral RNA packaging sequences are located in the 5′ leader sequence of the gRNA. The HIV-1 RNA packaging signal is located in the 5′ UTR after the major splice donor site and before the *gag* gene (Figure 3A) [134,147,148,149,150,151]. Because the sequences necessary for packaging are downstream of the splice donor site these sequences are only in unspliced RNA molecules; only the gRNA is specifically packaged over other RNAs. For HIV-2, the key sequences required for RNA packaging are located upstream of the major splice site (Figure 3A) [152,153]. Sequences between the major splice donor and the beginning of the Gag ORF have also been shown to play a role in HIV-2 gRNA packaging, but the effect on packaging when these sequences are deleted or mutated is less than when the sequences upstream of the splice donor are mutated [152,154]. The sequences downstream of the splice donor site have been reported to bind to HIV-2 Gag [155]. This RNA-protein interaction likely permits selectivity between spliced RNAs and the unspliced gRNA that is not encoded solely by sequences upstream of the splice donor site. The differences in the location of RNA packaging sequences in HIV-1 and HIV-2 relative to the splice donor site suggest potential differences in the mechanistic details of gRNA packaging, which could have implications for particle assembly and infectivity.

For HIV-1 and HIV-2, the RNA packaging signal contains four stem-loop structures (i.e., SL1, SL2, SL3, and SL4). These structures are the key sites for Gag-gRNA interactions [156,157]. All 4 stem-loops can independently bind Gag, and all four of the stem-loops have been shown to be important for RNA packaging [158,159,160,161,162,163]. Evidence supports the conclusion that SL3 has the most significant contribution to gRNA packaging, followed next by SL1 [108,134,160,164,165,166,167]. In particular, a 110 nt sequence in the gRNA from position 227–337 represents the core Gag binding domain [168]. The stem-loop structure of this region is mediated by a long-range interaction between a CU-rich region and nucleotides proximal to the AUG start codon [168]. In addition to RNA stem-loop structures, it has more recently become apparent that unpaired guanosine bases in the packaging signal contribute to specific Gag binding of gRNA, perhaps even more than SL1 and SL3 for HIV-1 as determined in cell-free studies using a shortened RNA containing the packaging signal [129,169]. Similar observations have been made for unpaired guanosine nucleotides in the full-length genome [161]. Unpaired guanosine nucleotides are also present at several locations in the packaging signal that have previously been identified to be primary HIV-1 Gag binding sites [161]. Mutation of a few unpaired guanosine nucleotides in one Gag binding site was found to cause a minor defect in gRNA packaging, but the combination of mutations at unpaired guanosine nucleotides of multiple Gag binding sites resulted in a synergistic reduction in gRNA packaging [163]. This suggests that multiple Gag molecules binding to gRNA are required for packaging. Furthermore, these observations imply functional redundancy in the RNA packaging mechanism. Unpaired guanosines in the HIV-2 RNA packaging signal have also been implicated in gRNA packaging [170], which suggests conservation of the RNA packaging mechanism. Other nucleotides have been identified that play a more secondary role in specific gRNA packaging, as they are not Gag binding sites. For the HIV-1 RNA packaging signal, the nucleotides at positions 226 and 227 have been reported to promote gRNA packaging efficiency by regulating RNA secondary structure of the packaging signal [171]. 

The HTLV-1 packaging signal has not been as extensively characterized. Bovine leukemia virus (BLV), a closely related deltaretrovirus, has provided insight into 5′ leader sequences involved in HTLV-1 RNA packaging. Two RNA stem-loop structures in the HTLV-1 *gag* gene that are homologous to stem-loops in BLV that were shown to be involved in gRNA packaging were able to be substituted in the BLV genome and allowed for RNA packaging (Figure 3A) [172]. This implies that these stem-loop structures are likely involved in HTLV-1 gRNA packaging. The contributions of other sequences involved in gRNA packaging for HTLV-1 such as those in the 5′ UTR are not well characterized.

### 4.2. Gag NC Determinants of Gag-gRNA Interaction

The RNA-protein interaction mediating packaging of gRNA is known to involve the NC domain of Gag. The NC domain of HIV-1 and HIV-2 Gag has been shown to bind the viral gRNA (Figure 3) [125,126,127,128,129,130,131,132,133,134]. The specific motifs in NC important for interaction with the gRNA include two ZnFs, each of which is formed by a cysteine-histidine (Cys-His) box, (Figure 3B) [144,158,169,173,174,175,176]. These similar yet distinct ZnFs are thought to contribute differently to viral gRNA packaging. While both are required, the N-terminal ZnF is thought to be more dominant and the two ZnFs are known to not be interchangeable [158,177,178,179,180,181]. A recent study more completely characterized the role of ZnFs in gRNA packaging. This report suggests that both ZnFs mediate Gag-gRNA interaction in the cytoplasm equally, but each ZnF contributed individually to Gag-gRNA accumulation at the plasma membrane with the more C-terminal ZnF being more crucial to this activity [114]. Specific NC residues involved in gRNA binding have been characterized, and include positively charged, basic amino acids within the ZnFs [178,179,182,183,184]. However, mutation of both ZnFs did not completely inhibit HIV-1 gRNA packaging, providing evidence that the Gag-gRNA interaction is mediated by other residues outside of the ZnFs [185]. Taken together, these observations suggest a model for gRNA packaging in which basic residues and ZnFs of HIV-1 Gag NC cooperate to select gRNA to be packaged into assembling viral particles.

The mechanism by which ZnFs promote binding to gRNA has been of great interest. An NMR spectroscopy study of the structure of HIV-1 NC in complex with SL3 of the HIV-1 gRNA packaging signal revealed that the ZnFs form hydrophobic clefts that bind to unpaired guanosines [186]. More recent work has described how ZnF metalation may also support that structure. Gag variants containing two Zn^2+^ molecules were shown to have a higher binding affinity to and selectivity for model gRNA when compared to Gag variants with one Zn^2+^ molecule [181]. These findings support that both ZnFs of Gag must be metallated by Zn^2+^ for gRNA packaging. It has been hypothesized that this permits gRNA packaging, as the structure of metallated ZnFs brings basic residues flanking these motifs closer together to form a basic region that could serve to select RNA. The binding of Gag to a model gRNA compared to binding to other viral RNAs has revealed how ZnFs may mediate specific interactions. In particular, it was observed that Gag binds to gRNA in a specific salt-independent manner selecting for gRNA over other RNAs in the cell [169]. ZnF metallation may also be important in differentiating the function of the NC domain within full-length Gag from that of NCp7, mature cleavage product of Gag encoded by the NC region. This is supported by a decreased affinity and selectivity of NCp7 for gRNA regions that promote packaging [181]. Notably, cleavage of Gag that creates NCp7 favors nonspecific nucleic acid interactions that allow NCp7 to coat the entire genome in mature virus particles. HIV-2 and HTLV-1 Gag have ZnF motifs similar to that of HIV-1 Gag (Figure 3B). Further study of the specific interaction of HIV-2 and HTLV-1 Gag and the respective gRNAs is needed to understand the mechanistic similarities in packaging retroviral gRNAs. 

### 4.3. Role of Other Gag Domains in Gag-gRNA Interaction

Other domains of Gag have been shown to contribute to Gag-gRNA interactions involved in retroviral gRNA packaging. HIV-1 NC, not in the context of the full-length Gag protein, can bind RNA with high-affinity, but without specificity, though NC is required for specificity and binding affinity in full-length Gag [157,181]. This suggests a role for other domains of Gag in packaging specificity. For HIV-1, the Gag domains SP1 and p6 have both been shown to be involved in selectively binding gRNA. The p6 domain is required for specific high-affinity binding to gRNA, while mutations in SP1 reduced HIV-1 gRNA packaging through a mechanism that is not fully understood [187,188,189]. It has been suggested that the p6 domain indirectly supports selective RNA binding through interaction with Gag NC ZnFs. This has been suggested to promote steric selection of gRNA by masking the positive charges of Gag NC. The deltaretroviral MA domain of Gag has been shown to potentially play an important role in gRNA packaging. The HTLV-1 and HTLV-2 MA proteins have higher nucleic acid binding affinity than HTLV-1 NC and can act as a nucleic acid chaperone [142,190]. Furthermore, the HTLV-2 MA specifically binds HTLV-2 gRNA with high-affinity [190]. For other deltaretroviruses, specific residues in MA have been shown to be required for gRNA packaging. The HIV-2 Gag MA domain also has been reported to have nucleic acid chaperone activity [191]. It is plausible that both MA and NC domains contribute to HIV-2 gRNA packaging. There is a paucity of virological studies to fully validate cell-free studies to date and it is an important direction for future investigations. The function of other Gag domains in gRNA packaging, as well as their roles in direct or indirect gRNA recognition, need further clarification as the role of Gag domains in gRNA packaging varies among retroviruses. These differences can provide deeper insights into the various mechanisms evolved by retroviruses. 

A recent study reported that HIV-1 CA and MA domains have a functional role in genome packaging as Gag multimerization and membrane binding, mediated by both the CA and MA domains, are required for genome packaging [192]. These observations support a model for HIV-1 gRNA packaging in which Gag proteins interact with the gRNA at the plasma membrane, multimerize, and lead to nucleation of the particle assembly complex.

### 4.4. Dimerization Initiation Site/Dimer Initiation Signal (DIS) Mediates gRNA-gRNA Interaction

The interaction of two gRNA molecules, or dimerization, is the second key interaction in gRNA packaging. Where gRNA dimerization occurs in the cell has been studied. For HIV-1, studies have suggested that dimerization occurs after the gRNA is exported from the nucleus, likely at or near the plasma membrane, but prior to gRNA packaging [104,193,194]. For example, a recent study tracked individual HIV-1 RNA molecules and found that individual gRNAs dimerize at the plasma membrane and do not arrive as dimers [194]. The RNA secondary structure can impact gRNA dimerization, given that the RNA structure involved in dimerization is located within the packaging signal [150,195]. In particular, HIV-1 has a palindromic sequence in the SL1 of the packaging signal that mediates dimerization, i.e., the dimerization initiation site (DIS) [196,197,198,199]. The key nucleotides mediating this interaction are located at the top of the SL1 hairpin loop (i.e., the kissing loop) (Figure 3C). Dimerization relies on the guanine quartets in the hairpin loop [200], where dimerization occurs through annealing of the palindromic sequences by a loop-loop interaction between two monomers with no extended duplex [196,201,202]. A second aspect of the dimerization mechanism involves intrastrand base pairs that form the stems of the hairpin loops and dissociate and reanneal to form a stable interstrand duplex [196]. The extended dimer conformation of dimerized HIV-1 gRNA molecules has been shown to be similar among various HIV-1 strains [203], suggesting a consistent model of dimerization among HIV-1 strains.

Similarities exist in the dimerization mechanisms for other human retroviruses. The HIV-2 DIS is highly similar to HIV-1 and is also a 6-mer palindromic sequence located in a hairpin loop within SL1 (Figure 3C) [204,205,206]. Another HIV-2 hairpin loop that may mediate dimerization is located in the trans-activation response (TAR) element [206,207]. Furthermore, a sequence in the HIV-2 primer binding site (PBS) is involved in dimerization [205,208,209]. Higher RNA concentrations were used in these studies, so the identified role of TAR and PBS in dimerization could be artifacts due to nonspecific interactions. During virus particle assembly, the tRNA^lys3^ binds the PBS, which changes the RNA structure and likely inhibits RNA dimerization. Though the PBS may permit gRNA dimerization, it would not do so in the context of gRNA packaging. More recent findings suggest it is also possible that the PBS was identified as a DIS because tRNA^lys3^ binding changes the overall gRNA structure in a way that promotes dimerization through the DIS identified in SL1 [210]. The HIV-1 and HIV-2 DIS hairpin loops are too distinct from one another to permit heterodimerization [211], suggesting that the likelihood of HIV-1 and HIV-2 recombination would be low. There have been two DIS sequences identified for HTLV-1, with both being palindrome-like sequences located within loop structures in the 5′ leader sequence upstream of the PBS (Figure 3C) [142,212,213,214]. One of these sites contains a structural motif that is different than the hairpin loops involved in HIV-1 and HIV-2 dimerization. The RNA forms a trinucleotide RNA loop that has been implicated in dimerization [215]. The differences in dimerization among deltaretroviruses and lentiviruses, and their implications, remain to be determined.

### 4.5. Regulation of gRNA Dimerization by RNA Conformation 

The conformation of the gRNA is important for mediating the interaction between two gRNA molecules. Two major RNA structural conformations, i.e., a long-distance interaction (LDI) conformation in which the DIS is inaccessible, and a branched molecular hairpin (BMH) conformation in which the DIS is exposed, have been observed [216]. For HIV-1, the LDI conformation is prompted by interactions between the polyA sequence and DIS hairpin motifs [216]. For HIV-2, the LDI structure forms through interaction of nucleotide bases upstream of the PBS and sequences in the vicinity of the Gag start codon [198,217,218]. For both HIV-1 and HIV-2, the BMH structure, which is composed of several intramolecular interactions, promotes dimerization by altering the gRNA structure such that the palindromic sequence of the hairpin loop is exposed [219,220,221,222,223]. Both the HIV-1 and HIV-2 BMH structures promote gRNA packaging, which implies a linkage in these two processes [146,220,224]. Taken together, these observations suggest a mechanism in which changes in RNA structure may serve as a molecular switch between processes associated with gRNA packaging—i.e., dimerization, NC binding, and viral protein translation. Among lentiviruses, this molecular switch, appears conserved, while little is known for deltaretroviruses like HTLV-1. 

Other sequences forming intramolecular interactions have been proposed to contribute to dimerization. In particular, sequences located within the major splice donor site and downstream sequences have been predicted to form intramolecular interactions that promote dimerization [196,225]. For HIV-2, an additional palindromic sequence (pal) located in the stem B region of SL1 was found to be necessary for dimerization [206,226,227,228]. This pal sequence has been shown to permit intramolecular interactions with upstream and downstream sequences in the 5′ UTR that are predicted to alter how the DIS is exposed [162,229,230,231]. A recent study reported a role for a key intramolecular interaction that is present only in the BMH conformation between sequences in the U5 region and in the vicinity of the Gag start codon [231]. Specifically, when sequences near the Gag start codon were replaced with sequences that form a stable hairpin structure, dimer stabilization was maintained. However, when the sequence was altered to sequester the sequence surrounding the Gag AUG, the U5:AUG interaction was prevented, which inhibited both dimerization and gRNA packaging [231]. This and other studies have confirmed the importance of intramolecular interactions in stabilizing gRNA dimers.

### 4.6. Role of Gag in Promoting gRNA Dimerization

Gag has been implicated in facilitating gRNA dimerization. A positive correlation between HIV-1 Gag concentration in cells and RNA dimer formation has been reported [194]. A link between NC binding and RNA dimerization has also been observed [195,218]. The mechanism by which NC binding promotes dimerization is presently unknown, but it is plausible that Gag binding to gRNA could stabilize dimers, or that the chaperone activity of NC may promote gRNA dimerization. In support of this, it has been observed that NC binding may enhance gRNA dimerization by stabilizing the BMH conformation [216]. However, it has also been demonstrated that gRNA can adopt a stable conformation with 5′ UTR intermolecular base pairing with the AUG start codon of the *gag* gene in the absence of NC domain of Gag as well as any RNA chaperone [232]. The mechanism for how Gag promotes dimerization remains an outstanding question in the field. It has also been observed that dimerization may occur between two Gag-gRNA duplexes, based on the observation that gRNA behavior is influenced by Gag near the plasma membrane [122]. This suggests that gRNA near the membrane interacts with Gag that is at low copy numbers [122].

### 4.7. Other RNA Sequences Contribute to gRNA Packaging: Secondary Packaging Signals

Other regions of retroviral genomes have been reported to contribute to gRNA packaging. While not required for packaging, the HIV-1 RRE sequence has been shown to enhance gRNA packaging, as has HIV-1 Rev, beyond the effect of exporting gRNA from the nucleus [231,233,234,235,236]. The effect of Rev-mediated enhancement of packaging was also demonstrated when a similar RNA was exported by NXF1, suggesting that Rev has a role in gRNA packaging beyond its role in mediating gRNA nuclear export [104,234]. The exact mechanism by which Rev enhances gRNA packaging is poorly understood, though it has been indicated to be indirect [230]. The interaction of the gRNA with Rev has been suggested to alter the RNP complex, or in general, make the gRNA more accessible for selection by Gag. The RRE sequence may contribute to long-distance interactions that stabilize RNA structures necessary for Gag-gRNA interactions. To date, there have not been any reports of a contribution of Rev/RRE in HIV-2 gRNA packaging [237]. Other genomic sequences potentially involved in gRNA packaging include a sequence in the HIV-1 Gag P1-P6 domain, as well as the Gag-Pol ribosomal frameshift site [238]. However, subsequent work has shown that the frameshift site is not required for packaging [239]. Differences in the experimental systems explain the differences in observations made.

The observed differences in gRNA packaging mechanisms among human retroviruses are further underscored by the differential ability to cross-package or co-package gRNAs. In gRNA cross packaging, the gRNA of one virus is incorporated into the virions of other viruses; gRNA co-packaging is the incorporation of a gRNA from one virus with a gRNA from another virus. Retrovirus gRNA cross packaging is not universal [240]. In particular, non-reciprocal gRNA packaging between HIV-1 and HIV-2 has been observed, where HIV-1 Gag-Pol can package HIV-2 gRNA, but HIV-2 cannot cross package HIV-1 gRNA [241]. The cross packaging and co-packaging of HTLV-1 with human lentiviruses have not been reported to date. Nonreciprocal cross packaging and co-packaging imply differences in gRNA packaging for retroviruses, which may include differences in binding sites or other aspects of gRNA packaging, including gRNA dimerization and/or translocation to particle assembly sites.

### 4.8. RNA Selection for gRNA Packaging Is Regulated on a Transcriptional Level

Whether a retroviral gRNA is utilized for packaging or for protein translation is a key aspect of the retrovirus lifecycle that has attracted great interest for many years. One favored model has been that there is a functional separation of gRNA for packaging and translation (i.e., separate pools). Inhibition of transcription does not reduce HIV-1 or HIV-2 gRNA packaging more rapidly than reductions that occur in cytoplasmic RNA levels [242,243,244]. Furthermore, inhibition of nuclear export by leptomycin B was not found to affect HIV-1 and HIV-2 gRNA packaging [243]. Together, these observations suggest that there is no functional separation of gRNAs into pools because the rate of decrease in gRNA packaging and translation of viral proteins matched the decrease in abundance of cytoplasmic RNAs. More recent studies have indicated that gRNA packaging is regulated at a transcriptional level, based on the observation that several versions of the same transcript are observed with varying numbers of 5′ capped guanosines [245]. Transcripts with a single capped guanosine are enriched in virions, while transcripts with two or three capped guanosines are enriched on polysomes [245,246]. This differential capping mechanism has been shown to alter the transcript structure in a way that correlates with changes in function. In particular, for gRNA with a single capped guanosine, the dimeric multi-hairpin conformation was promoted, resulting in an inaccessible cap that prevents interaction with eIF4E [247]. The addition of two or three capped guanosines forms a structure that will expose the cap, which favors RNA translation [247]. Another study supported this model by demonstrating that HIV-1 gRNA cap exposure can inhibit gRNA packaging [248]. Whether gRNA is selected for packaging in *cis* or in *trans* by Gag has been studied, and it was found that HIV-1 gRNA that cannot be translated was still packaged into virions [230,249]. Furthermore, it was reported that HIV-1 Gag selectively packages non-translated gRNA [250]. These observations imply that *cis* packaging is not a requirement for HIV-1 gRNA packaging. The HIV-2 gRNA packaging has also been shown to be random, implying that *cis* packaging is not required [249,251,252,253]. The fact that *cis* packaging is not required for gRNA packaging supports the conclusion for functional separation of gRNA for human retroviruses. 

## 5. Conclusions

Retroviral gRNA packaging represents a crucial step in the production of infectious virus particles and studies of this process provide greater insights into retrovirus replication. To date, knowledge regarding retrovirus gRNA packaging has come mainly from studies of HIV-1. Many details of gRNA packaging known for HIV-1 are poorly understood for closely related human retroviruses, i.e., HIV-2 and HTLV-1. Thus, comparative analyses of HIV-2 and HTLV-1 gRNA packaging would provide deeper insights into the details of gRNA packaging by revealing the conserved or novel mechanisms. The mechanism of gRNA nuclear export represents an important aspect of unspliced RNA molecule translocation to the cytoplasm. Viral elements essential for gRNA nuclear export have been defined for all three human retroviruses, but cellular proteins involved in gRNA nuclear export have, to date, only been characterized for HIV-1. Viral gRNA-protein interactions required for gRNA encapsidation and dimerization are well characterized, particularly for HIV-1 and HIV-2. A particular knowledge gap in the field involves the dynamics of gRNA packaging—particularly how the gRNA translocates to the plasma membrane. While various studies have identified cellular components that influence gRNA translocation to the plasma membrane, the mechanistic details remain unclear. The temporospatial dynamics of the gRNA and Gag are lacking beyond those studies at limited timescales and at various subcellular localizations. To date, studies of gRNA translocation to virus assembly sites have almost exclusively focused on HIV-1. Addressing these knowledge gaps will improve our understanding of the complex process of genome recognition and gRNA packaging.

## Figures and Tables

**Table 1:** Summary of cellular proteins involved in export of retroviral gRNA from the nucleus.

Protein	Function in gRNA Nuclear Export
CRM1	Major nuclear export receptor
Ran	G protein, Ran GTP hydrolysis cycle drives export of CRM1 complex
DDX1	Nucleates Rev oligomerization on RRE
DDX3	Restructures gRNA for translocation through NPC
Nup62, Nup98, Nup124, Nup153	Nucleoporins that have been identified as part of the NPC involved in gRNA nuclear export
PACS1	Nucleocytoplasmic shuttle protein that interacts with Rev-RRE-CRM1 complex
ANP32A/B	Mediate export of viral RNAs via interaction with Rev-RRE-CRM1 complex
eIF5a	Mediates Rev-RRE-CRM1 interaction with NPC
Rab/hRIP	Interacts with CRM1
UPF1	Nucleocytoplasmic shuttle protein that interacts with Rev-RRE-CRM1 complex
MOV10	Rev cofactor
Staufen2	Regulates Rev nuclear export
Tat-SF1	Interacts with gRNA and promotes nuclear export
